# Virucidal Activity of Different Mouthwashes against the Salivary Load of SARS-CoV-2: A Narrative Review

**DOI:** 10.3390/healthcare10030469

**Published:** 2022-03-03

**Authors:** Alvaro Garcia-Sanchez, Juan-Francisco Peña-Cardelles, Angel-Orión Salgado-Peralvo, Flor Robles, Esther Ordonez-Fernandez, Steve Ruiz, Dániel Végh

**Affiliations:** 1 Department of Oral Health and Diagnostic Sciences, School of Dental Medicine, University of Connecticut Health, Farmington, CT 06030, USA; 2 Department of Health Sciences, Rey Juan Carlos University, 28040 Madrid, Spain; 3 Oral and Maxillofacial Surgery Department, School of Dental Medicine, University of Connecticut Health, Farmington, CT 06030, USA; 4 Department of Prosthodontics, School of Dental Medicine, University of Connecticut Health, Farmington, CT 06030, USA; 5 Department of Stomatology, Faculty of Dentistry, University of Seville, 41009 Seville, Spain; orionsalgado@hotmail.com; 6 Division of General Dentistry, School of Dental Medicine, University of Connecticut Health, Farmington, CT 06030, USA; roblesmijangos@uchc.edu (F.R.); eordonezfernandez@uchc.edu (E.O.-F.); sruiz@uchc.edu (S.R.); 7 Department of Prosthodontics, Semmelweis University, 1085 Budapest, Hungary; vegh.daniel.official@gmail.com; 8 Department of Dentistry and Oral Health, Division of Oral Surgery and Orthodontics, Medical University of Graz, 8010 Graz, Austria

**Keywords:** COVID-19, SARS-CoV-2, mouthwashes, aerosols, chlorhexidine, povidone-iodine, cetylpyridinium chloride, hydrogen peroxide, colony-forming units

## Abstract

The saliva of COVID-19-confirmed patients presents a high viral load of the virus. Aerosols generated during medical and dental procedures can transport the virus and are a possible causative agent of cross-infection. Since the onset of the pandemic, numerous investigations have been attempting to mitigate the risk of transmission by reducing the viral load in saliva using preprocedural mouthwashes. This study aims to review the most up-to-date in vitro and in vivo studies investigating the efficacy of different mouthwashes on reducing the salivary viral load of SARS-CoV-2, giving particular attention to the most recent randomized control trials published.

## 1. Introduction

Severe acute respiratory syndrome coronavirus 2 (SARS-CoV-2) or COVID-19 was observed originally in December 2019 in Wuhan (China) [1]. Patients infected with COVID-19 presented involvement of multiple body systems and organs, including the kidneys, blood vessels, nervous system, heart, lung, gastrointestinal tract, and liver [2]. Numerous studies demonstrated that this virus can produce diverse sequelae in survivors, has considerable mortality and a severe socioeconomic impact on society [2,3].

There is evidence that SARS-CoV-2 can be transmitted by direct contact, droplets, and fomites, and by airborne transmission [4,5,6]. On 24 November 2021, South Africa reported the identification of the SARS-CoV-2 Omicron variant [7]. Since then, cases of this variant of COVID-19 have been increasing exponentially all over the world [7]. Although the initial data show that this variant is less severe, there are concerns over the high transmissibility, virulence, and an increased risk of infection, especially through airborne transmission [8,9].

Aerosols are defined as inspirable particles of liquid or solid in a gas [10]. They are composed of droplet nuclei of ≤5 μm in diameter and can be suspended in the air for hours. Medical and dental procedures that generate aerosols can lead to the transmission of SARS-CoV-2 to healthcare providers present in the procedure. The saliva of COVID-19 positive patients contains a high viral load of the virus, with the highest viral load in the first week after the onset of symptoms [11,12].

Since the beginning of the pandemic, there have been several investigations on the in vitro efficacy in the reduction of the salivary viral load using different mouthwashes. However, in vivo studies are necessary to confirm the validity of their findings. In the last few months, numerous randomized controlled trials (RCTs) about this topic have published their results.

Therefore, this review aims to show the most up-to-date and efficient methods of reduction of salivary viral load of SARS-CoV-2 using mouthwashes, with an emphasis on the latest RCTs available.

## 2. Material and Methods

The search was conducted in four different electronic databases: MedLine (via PubMed), SCOPUS, the Cochrane Library database, and the Web of Science (WoS).

The search strategy was carried out by two authors independently (A.G.-S. and A.-O.S.-P.). There were no time restrictions and it was updated to January 2022. The search was limited to English-language studies. MeSH (Medical Subjects Headings) terms, keywords, and other free terms were used with Boolean operators (OR, AND) to combine searches: (‘mouthwash’ OR ‘oral rinse’ OR ‘mouth rinse’ OR ‘povidone iodine’ OR ‘chlorhexidine chloride’ OR ‘hydrogen peroxide’ OR ‘cetylpyridinium chloride’ OR ‘essential oil’ OR ‘phthalocyanine derivatives’ OR ‘ethanol’ OR ‘citrox’ OR ‘Listerine’) AND (‘COVID-19’ OR ‘SARS-CoV-2’ OR ‘SARS’). The search in different databases followed their specific syntax rules. We also searched articles present in the reference lists of the resulting articles that were within the scope of this review.

## 3. Results

We selected 20 articles out of 1121 after deletion of the non-English literature, articles where the reduction of the salivary load of SARS-CoV-2 was not the outcome investigated, and opinion articles. The articles included were 11 in vitro studies, 1 clinical pilot study and 8 RCTs. Mouthwashes evaluated in the articles were chlorhexidine (CHX), povidone-iodine (PVP-I), hydrogen peroxide (H_2_O_2_), cetylpyridinium chloride (CPC), beta-cyclodextrin + Citrox^®^, ethanol + essential oils and iota-carrageenan (IC). Most of the articles tested more than one solution and the control or placebo solution was predominately distilled water. The most common mouthwash solution studied was PVP-I (present in 57% of the included studies), followed by CHX (47%), and H_2_O_2_ (24%). The solution most commonly studied in the RCTs included was CHX (67%), followed by PVP-I (56%), and H_2_O_2_ (22%). A summary of the findings of the included in vitro studies is described in Table 1.

There was a large discrepancy between the sample sizes of the RCTs. The number of patients recruited in the RCTs ranged from 36 to 294. All of these studies had rinsing times between 30 s and 1 min. There was some heterogenicity in the concentrations of the solutions. PVP-I concentrations ranged from 0.5 to 2%; CHX concentrations ranged from 0.12 to 0.2%; and H_2_O_2_ concentrations ranged from 1 to 1.5%. The other mouthwashes studied had the same concentrations through all of the studies included. A summary of the findings of the included in vitro studies is described in Table 2.

## 4. Discussion

Procedures with high-speed devices, intubation and extubation procedures, bronchoscopy, cardiopulmonary resuscitation, and high-flow oxygen therapy, are among the list of medical procedures that pose a risk of spreading COVID-19 in medical settings by creating aerosols [33]. In addition, dentists are one of the professions that have the highest risk of infection of COVID-19 due to the proximity with the patients’ oral cavities and the numerous aerosols generating procedures performed routinely [34]. Saliva and blood are the main components for viral spread, therefore, procedures that generate aerosols should be minimized [35]. Since the onset of the pandemic, personal protective equipment has been one the most important measures to prevent the transmission in medical and dental settings, but recently the emphasis has been placed on the use of preprocedural rinses to reduce the viral load in saliva.

Preoperative rinses reduce the number of microorganisms in the oral cavity and colony-forming units in dental aerosols [36]. Multiple associations recommended the use of preprocedural rinses before oral procedures [37,38,39]. Several in vitro and more recently in vivo studies have evaluated the efficacy of different mouthwashes to reduce the salivary viral load of SARS-CoV-2.

### 4.1. Chlorhexidine

Chlorhexidine is a safe and effective antiseptic solution with broad antiseptic activity. The mechanism of action increases the permeability of the bacterial cell, causing its lysis [40]. It is widely used in dentistry, predominantly to reduce dental plaque and to treat periodontal disease [41,42].

A study by Jain et al. [13] evaluated the in vitro efficacy of CHX at concentrations 0.2% and 0.12%. They found that the virucidal activity against COVID-19 was >99.99% at a concentration of 0.2% and 99.99% at a concentration of 0.12% at both 30 and 60 s contact times. Xu et al. [14] had similar results; however, the contact time in their study was 30 min, which would not be practical for clinical settings. On the other hand, Meister et al. [15] evaluated the log reduction factor of various compounds and found that CHX had reductions of <2 logs at both concentrations, which was less effective than the other solutions included in the study.

There are multiple recent RCTs performed evaluating the virucidal activity of CHX against salivary SARS-CoV-2. A study by Costa et al. [24] evaluated the efficacy of 0.12% CHX at 5 and 60 min after rinsing for 1 min. They found that there was a significant reduction (72%) in the salivary load at both 5 and 60 min after rinsing compared with the control. A study by Seneviratne et al. [25] showed that the reduction in viral load using 0.2% CHX was significantly lower compared with the one achieved with 0.5% PVP-I. Subsequently, a study by Eduardo et al. [26] demonstrated a significantly reduced viral load using 0.12% CHX after 30 and 60 min compared with the control group, but the reduction was lower than those seen in the H_2_O_2_ and CPC + zinc mouthwashes at 30 min.

Furthermore, a RCT by Elzein et al. [27] did not find a significant difference between 0.2% CHX and 1% PVP-I, and both were effective against salivary SARS-CoV-2. On a similar note, a RCT by Chaudhary et al. [28] evaluated the effectiveness of 0.12% CHX at 15 and 45 min after rinsing and found reductions of 80–89%, similar to the other mouthwashes evaluated (H_2_O_2_ and PVP-I).

When evaluating the efficacy of 0.12% CHX in the oropharynx, SARS-CoV-2 was eliminated in 62.1% of patients who used the oral rinse and 86% of patients when combined with an oropharyngeal CHX spray [29]. In contrast, a study by Ferrer et al. [30] evaluating different mouthwashes showed no statistically significant changes in salivary viral load after the use of any of the mouthwashes, including 0.12% CHX.

Chlorhexidine has been widely studied to reduce the salivary load of SARS-CoV-2. In general, the results show that it is safe and potentially useful as a preprocedural mouthwash; however, the net amounts of reduction were lower than other compounds in various studies.

### 4.2. Povidone-Iodine

PVP-I is composed of iodine and the water-soluble polymer polyvinylpyrrolidone. It disrupts several metabolic pathways and disorganizes the cell wall, eliminating the virus [43]. The common use of PVP-I in mouth rinse has no deleterious health effects [44]. However, its use is contraindicated in patients allergic to iodine or thyroid disease, and in pregnancy [45]. The American Dental Association, Centers for Disease Control and Prevention, and the Australian Dental Association recommended the use of a preprocedural 0.2% PVP-I mouth rinse to decrease the risk of SARS-CoV-2 transmission [37,38,39].

Hassandarvish et al. [16] evaluated the in vitro virucidal efficacy of PVP-I at various concentrations and contact times. Virucidal activity of >5 log_10_ was reported at 15 s with 1% PVP-I and at 30 s with 0.5% PVP-I. Similar studies found virucidal activities of >4 log 10 at 15 [17], 30 [18], and 60 [13,19] s. A study by Xu et al. [14] found a virucidal activity of >99.9% with a contact time of 30 min. Another study evaluating PVP-I as a nasal antiseptic rinse demonstrated complete inactivation of the virus by concentrations as low as 0.5% after 15 s of contact [20].

Several RCT studies have evaluated the efficacy of PVP-I against SARS-CoV-2 in saliva. One study evaluated the effectiveness of 0.5% PVP-I on reducing the viral load at various intervals and found that at 15 min, there was a reduction of 61%, whereas at 45 min the reduction was 97% [28]. Seneviratne et al. [25] evaluated saliva samples after using 0.5% PVP-I at 5 min, 3 h, and 6 h after rinsing. The results showed high virucidal activities at 5 min and 3 h post-rinsing, but it was only statistically significant at 6 h compared to distilled water.

A study by Elzein et al. [27] evaluated the reduction in salivary load in 61 patients 5 min after rinsing with 1% PVP-I for 30 s. A significant difference was noted between the delta cycle threshold (Ct) of distilled water (control group) and 1% PVP-I. On the other hand, Ferrer et al. [30] evaluated the use of 2% PVP-I on the reduction of salivary viral load and found no statistically significant changes in salivary viral load after the use of the different mouthwashes, including PVP-I.

PVP-I has also been widely studied both in vitro and in vivo. In general, it has a great success in reducing the salivary load of SARS-CoV-2 as a preprocedural rinse. It is safe and it presents a low number of contraindications. Contact times ranging from 30 s to 1 min are ideal for clinical practice. This molecule has the potential to be one of the most effective preprocedural mouthwashes against SARS-CoV-2.

### 4.3. Hydrogen Peroxide

H_2_O_2_ is a widely used antimicrobial agent and it is effective against several viruses including adenovirus, rhinovirus, myxovirus, and influenza A [46].

An in vitro study reported log reductions of the viral load using H_2_O_2_ of < 1 at 30 s. This reduction was significantly lower than all other mouthwashes studied (PVP-I, CHX, Ethanol + essential oils) [15]. On the other hand, Xu et al. [31] reported a kill rate of > 99.9%; however, the contact time was 30 min.

A prospective clinical pilot study found that 1% H_2_O_2_ does not reduce the intraoral viral load in COVID-19 positive patients [31]. However, a RCT evaluating the efficacy of multiple types of mouthwash found that rinsing with H_2_O_2_ resulted in a significant reduction of salivary viral load up to 30 min after rinsing, but the reduction at 60 min was not significant [26]. Similarly, a RCT by Chaudhary et al. [28] evaluated the viral load of multiple types of mouthwash at 15 and 45 min after rinsing. The use of a 1% H_2_O_2_ mouthwash resulted in significant reductions of 80–89%, similar to the other mouthwashes evaluated (CHX and PVP-I). On the other hand, a study by Ferrer et al. [30] with a sample of 84 patients evaluated the use of 1% H_2_O_2_ for the reduction of salivary viral load. There were no statistically significant changes in virucidal effectiveness after the use of the H_2_O_2_ mouthwash.

### 4.4. Cetylpyridinium Chloride

CPC is a quaternary ammonium compound used in over-the-counter mouthwashes with broad antimicrobial activity and it also acts against viral capsids [47].

A RCT by Seneviratne et al. [25] concluded that the salivary viral load of SARS-CoV-2 decreased significantly with the use of CPC mouthwash at 6 h, comparable with the reduction using PVP-I. Similarly, Eduardo et al. [26] found a significant reduction in viral load for up to 60 min after rinsing when using CPC + Zinc. A study by Rodríguez-Casanovas et al. [21] evaluated a commercial mouthwash containing a combination of 0.05% CPC + 0.2% D-limonene. They observed a statistically significant reduction of about 6 logs in the viral load compared with the control. On the other hand, Ferrer et al. [30] did not find any significant changes in the salivary load after using 0.07% CPC or any other solutions studied.

### 4.5. Beta-Cyclodextrin + Citrox^®^

This is a commercially available mouthwash composed of beta-cyclodextrin (excipient) and Citrox^®^ (flavonoids).

A RCT by Carrouel et al. [32] studied the efficacy of this compound on reducing the salivary viral load in COVID-19 positive patients. Participants were instructed to rinse three times per day for 7 days. They found that the use of this compound had a significant beneficial effect on reducing viral load 4 h after the initial dose, but the reduction was moderate for long-term effects.

### 4.6. Ethanol

Ethanol serves as an excipient in numerous oral rinses. It inactivates enveloped viruses at concentrations higher than considered safe for oral use (≥70%). When used as an excipient, the concentrations range between 14 and 27% [25,48,49].

Two studies investigated the in vitro efficacy of ethanol as a positive control against SARS-CoV-2 [37,48]. In one study, the 70% ethanol was unable to completely inactivate the virus after 15 s but was effective at 30 s of contact [22]. On the other hand, another study found that an intervention time of 15 s is enough to eliminate the virus [17].

Two in vitro studies evaluated the efficacy of commercially available products containing essential oils and ethanol (i.e., Listerine) [14,15]. These compounds were as effective as PVP-I in reducing the viral titer (≥3.11 log_10_), constituting a significant reduction compared to the control group.

### 4.7. Iota-Carrageenan

IC is a derivative from red marine algae with virucidal activity in vitro against rhinovirus, herpesviruses, and influenza A [50,51]. An in vitro study by Bansal et al. [23] found that concentrations easily achievable by nasal and nebulization formulations (600 μg/mL, 60 μg/mL, and 6 μg/mL), demonstrated statistically significant reductions in virus titers when compared with untreated controls.

### 4.8. Limitations of this Review

COVID-19 is a disease that is continuously being investigated, and multiple RCTs in progress at this moment are evaluating the use of different mouthwashes. Our findings must be interpreted with caution and further investigations must be carried out soon. Further in vitro studies evaluating potential new molecules and additional RCTs are essential to demonstrate the safety and effectiveness of different mouthwashes.

## 5. Conclusions

Within the limitations of this review, at present, PVP-I, CHX, and CPC are successful in reducing the salivary load of SARS-CoV-2 and could be used routinely to prevent the risk of cross-infection in medical and dental settings. Other mouthwashes have favorable initial results, but more studies and clinical trials must prove their efficacy and safety before they are routinely used.

## Figures and Tables

**Table 1:** Summary of the in vitro studies.

Author/ Year	Viral Culture	Solutions	Contact Time	Conclusions
Jain et al. [13] (2021)	SARS-CoV-2 stock using Vero E6 cell line	0.12% and 0.2% CHX ^1^ and 1% PVP-I ^2^	30 and 60 s ^3^	Both solutions achieved ≥99.9% inactivation at 30 and 60 s contact times
Xu et al. [14] (2021)	SARS-CoV-2 in Vero E6 cells and pseudotyped SARS-CoV-2 virus	Listerine Original^®^, 0.12% CHX, and 1.5% H_2_O_2_ ^4^	30 min ^5^	All solutions completely inactivated the virus
Meister et al. [15] (2020)	SARS-CoV-2 using Vero E6 cells	1.5% H_2_O_2_, 0.2% CHX, 0.15% BC ^6^ + 0.35% DC ^7^, 0.5% PVP-I, Listerine Cool Mint^®^, Octenident^®^, and ProntOral^®^	30 s	0.15% BC + 0.35% DC, 0.5% PVP-I, and Listerine^®^ presented significant virucidal activities of ≥99%
Hassandarvish et al. [16] (2020)	SARS-CoV-2 virus stock using Vero E6 cells	0.5% and 1% PVP-I	15, 30, and 60 s	Both concentrations demonstrated ≥99.99% virucidal activities at the different contact times
Bidra et al. [17] (2021)	SARS-CoV-2 in Vero 76 cells	0.5%, 1.25%, and 1.5% PVP-I; 1%, 5%, and 3% H_2_O_2_.	15 and 30 s	All concentrations of PVP-I inactivated the virus at both contact times, while H_2_O_2_ was minimally effective at both concentrations
Anderson et al. [18] (2020)	SARS-CoV-2 propagated in Vero E6 cells	0.45%, 1%, 7.5%, and 10% PVP-I	30 s	All four concentrations resulted in virucidal activities of ≥99.99%
Pelletier et al. [19] (2021)	SARS-CoV-2 in Vero 76 cells	1%, 2.5%, and 5% PVP nasal spray; 1%, 1.5% and 3% PVP oral rinse	60 s	All solutions tested completely inactivated the SARS-CoV-2
Frank et al. [20] (2020)	SARS-CoV-2 in Vero 76 cells	0.5% 1.25%, and 2.5% PVP-I	15 and 30 s	PVP-I at all concentrations completely inactivated SARS-CoV-2 within 15 s
Rodriguez-Casanovas et al. [21] (2021)	SARS-CoV-2 from positive nasopharyngeal swabs	8% PVP-I, 0.3% D-limonene, 0.1% and 0.07% CPC ^8^, 10% CHX, 0.12% CPC + 0.05% CHX, Listerine^®^ Zero Alcohol, 0.12% and 0.2% CHX, 0.05% NaF ^9^ + 0.075% CPC, 0.2% D-limonene, and 0.05% CPC	60 s	0.2% D-limonene + 0.05% CPC compound reduced the viral load >99.999%, while the other solutions did not show a reduction in viral load
Bidra et al. [22] (2020)	SARS-CoV-2 in Vero 76 cells	0.5%, 1%, and 1.5% PVP-I	15 and 30 s	All concentrations resulted in a complete inactivation of SARS-CoV-2 at 15 s
Bansal et al. [23] (2021)	SARS-CoV-2 in Vero E6 cells	Concentrations of 600 μg/mL, 60 μg/mL, 6 μg/mL, and 0.6 μg/mL of IC ^10^ in NaCl ^11^ solution	48 h ^12^	All concentrations demonstrated statistically significant reductions of the viral load of SARS-CoV-2

^1^ CHX, chlorhexidine, ^2^ PVP-I, povidone iodine, ^3^ s, seconds, ^4^ H_2_O_2_, hydrogen peroxide, ^5^ min, minutes, ^6^ BC, benzalkonium chloride, ^7^ DC, dequalinium chloride, ^8^ CPC, cetylpyridinium chloride, ^9^ NaF, sodium fluoride, ^10^ IC, iota-carrageenan, ^11^ NaCl, sodium chloride, ^12^ h, hours.

**Table 2:** Summary of the clinical trials included.

Author/ Year	Sample Size	Time of Testing	Intervention/Duration of Rinses		Conclusions
			Control Group	Test Group(s)	
Costa et al. [24] (2021)	100	RT-PCR ^1^ at baseline, 5 and 60 min ^2^ after rinsing	Placebo (inactive substance)	15mL ^3^ of 0.12% CHX ^4^/ 1 min	There was a significant reduction in the salivary load at both 5 and 60 min after rinsing compared with the control. There was a reduction in the load of SARS-CoV-2 in 72% of the volunteers using CHX vs. 30% in the control group
Seneviratne et al. [25] (2020)	36	Saliva samples for RT-PCR taken at baseline, 5 min, 3 and 6 h ^5^ after rinse	Placebo (water)/ 30 s ^6^	0.5% PVP-I ^7^, 0.2% CHX, 0.075% CPC ^8^/30 s	There were no differences in the reduction of salivary load in all intervention groups. PVP-I and CPC showed a significant reduction at 6 h and 6h and 5 min when compared with the control group
Eduardo et al. [26] (2021)	60	Saliva samples for RT-PCR collected at baseline, 30 and 60 min after rinse	Placebo (distilled water)/ 1 min	0.075% CPC + 0.28% Zn ^9^ (30 s), 1.5% H_2_O_2_ ^10^ (1 min), 0.12% CHX ^9^ (30 s), or 1.5% H_2_O_2_ + 0.12% CHX (1 min + 30 s)	CPC + Zn and CHX were effective in reducing the salivary viral load 60 min post-rinse. H_2_O_2_ was effective only at 30 min post-rinse
Elzein et al. [27] (2021)	61	Saliva was collected at baseline and 5 min after rinsing	Placebo (distilled water) /30 s	1% PVP-I and 0.2% CHX/ 30 s	The Ct ^11^ of the intervention groups (CHX 0.20% and 1% PVP-I) was significantly different compared to the control group
Chaudhary et al. [28] (2021)	40	Two samples of saliva taken at 15 and 45 min post-rinse	Placebo (normal saline)/ 60 s	1% H_2_O_2_, 0.12% CHX, 0.5% PVP-I. Rinsed with 15 mL/60 s	All 4 mouthwashes reduced the salivary load by 61–89% at 15 min and by 70–97% at 45 min
Huang et al. [29] (2021)	294	Oropharyngeal swab collected 4 days post-rinse for RT-PCR	Untreated control group	0.12% CHX/ 30 s 2/day and 0.12% CHX/30 s 2/day + oropharyngeal spray (1.5 mL) 3 times daily	SARS-CoV-2 was eliminated from the oropharynx in 62.1% of patients who used CHX as an oral rinse, vs. 5.5% of the control group. In the combination group, 86.0% eliminated oropharyngeal SARS-CoV-2 vs. 6.3% of control patients
Ferrer et al. [30] (2021)	84	RT-PCR at baseline, 30, 60 and 120 min after mouth rinsing	Placebo (distilled water)/ 1 min	2% PVP-I, 1% H_2_O_2_, 0.07% CPC, 0.12% CHX/ 1 min	None of the mouthwashes evaluated presented a statistically significant change in the salivary viral load
Gottsauner et al. [31] (2020)	12	RT-PCR at baseline and 30 min after intervention	0.9% NaCl/30 s	1% H_2_O_2_/30 s	No statistically significant differences between baseline viral load and 30 min after rinsing with 1% H_2_O_2_
Carrouel et al. [32] (2021)	176	Rinsed 3 times daily. Saliva collected at baseline, 1 h before the two following rinses, and last taken 1 h after the 2nd rinse	Placebo (distilled water)/ 1 min	30 mL of 0.1% beta-cyclodextrin and 0.1% Citrox^®^ rinse (CDCM^®^)/ 1 min	CDCM^®^ was effective at 4 h post-rinse. At day 7, only a modest virucidal activity was observed

^1^ RT-PCR, reverse-transcription polymerase chain reaction; ^2^ min, minutes; ^3^ mL, milliliters; ^4^ CHX, chlorhexidine; ^5^ h, hour(s); ^6^ s., seconds; ^7^ PVP-I, povidone iodine; ^8^ CPC, cetylpyridinium chloride; ^9^ Zn, zinc; ^10^ H_2_O_2_, hydrogen peroxide; ^11^ Ct, cycle threshold.

## Data Availability

Not applicable.
